# Ameliorating Effects of *Bifidobacterium longum* subsp. *infantis* FB3-14 against High-Fat-Diet-Induced Obesity and Gut Microbiota Disorder

**DOI:** 10.3390/nu15194104

**Published:** 2023-09-22

**Authors:** Ruixin Kou, Jin Wang, Ang Li, Yuanyifei Wang, Bowei Zhang, Jingmin Liu, Yi Sun, Shuo Wang

**Affiliations:** Tianjin Key Laboratory of Food Science and Health, School of Medicine, Nankai University, Tianjin 300350, China; kourx@mail.nankai.edu.cn (R.K.); wangjin@nankai.edu.cn (J.W.); angli@mail.nankai.edu.cn (A.L.); wangyyf163@163.com (Y.W.); bwzhang@nankai.edu.cn (B.Z.); liujingmin@nankai.edu.cn (J.L.); 9920220161@nankai.edu.cn (Y.S.)

**Keywords:** *Bifidobacterium longum* subsp. *infantis*, high-fat diet, obesity, gut microbiota, lipid metabolism, liver function

## Abstract

Obesity has emerged as one of the most prevalent chronic diseases worldwide. Our study was conducted to investigate the anti-obese potential of novel probiotic *Bifidobacterium longum* subsp. *infantis* FB3-14 (FB3-14) and the underlying molecular mechanisms in high-fat diet (HFD)-fed mice. The results demonstrated that an 8-week FB3-14 intervention significantly suppressed the HFD-induced body and fat weight gain and abnormal alterations of the serum lipid parameter, restoring the levels of cholesterol (4.29 mmol/L) and low-density lipoprotein cholesterol (3.42 mmol/L). FB3-14 treatment also attenuated adipocyte expansion, hepatic injury, and low-grade systemic inflammation and restored the expressions of lipid-metabolism-related genes, including *Hsl*, *Leptin*, and *Adiponectin*. Furthermore, FB3-14 was observed to reduce the Firmicutes/Bacteroidetes ratio in obese mice; increase the abundance of *Akkermansia muciniphila*, *unclassified_Muribaculaceae*, *Lachnospiraceae_NK4A136_group*, and *Bifidobacterim*; and upregulate G protein-coupled receptor41 associated with higher levels of butyric acid. These results indicate the protective effectiveness of FB3-14 in HFD-driven obesity and gut microbiota disorders, highlighting the promising potential of FB3-14 as a functional nutrition supplement.

## 1. Introduction

Along with the development of food consumption toward higher quality and excess nutrition, obesity induced by a high-fat diet (HFD) has become the most prevalent chronic disease worldwide, severely endangering human health. Excessive lipid accumulation is strongly associated with the progression of metabolic complications, including hyperglycemia, cardiovascular disease (CVD), steatohepatitis, and nonalcoholic fatty liver disease (NAFLD) [1]. Obesity is often typical of dyslipidemia, systemic inflammation, and intestinal dysfunction [2]. Previous studies have demonstrated the controlling effects of the gut microbiota on organism metabolism in germ-free mice and revealed its vital impact on the pathogenesis of obesity [3,4]. The variation and plasticity of gut microbiota are determined by both internal genetic and external factors, and gut microbiota dysbiosis induced by HFD was shown to be a cause of obesity rather than a result [5]. In view of the challenges of polypharmacy, reduced compliance, and side effects of synthetic drugs in the treatment of obesity for humans [6,7], the natural and promising alternatives targeting the gut microbiome are critical to obesity prevention.

Manipulating the intestinal microbiota with probiotics is considered a potential strategy to improve metabolic syndrome [8]. Among them, Bifidobacterium, as one of the principal symbiotic members in the gut, was found to be significantly enriched in the anti-obesity treatment of prebiotics (such as fiber and polyphenols), suggesting the correlation between strains and anti-obesity effects [9,10,11]. Additionally, certain edible probiotics belonging to the species *B. adolescentis*, *B. animalis*, *B. bifidum*, and *B. longum* have been widely used as nutritional supplements contributing to human health [12]. Bifidobacteria were also reported to reduce weight gain, inhibit serum and liver total cholesterol and triglycerides, improve fat deposition and glucose homeostasis, and notably, affect energy and/or lipid metabolism in a strain-dependent manner [12,13,14]. Although probiotic interventions have been recognized to alleviate metabolic disorders by altering the gut microbiota or its metabolites [15], given the presence of commensal bacteria, there is still a lack of studies on the anti-obesity roles of *B. longum* subsp. *infantis*. Particularly, the benefits of *Bifidobacterium* delivery on intestinal microecology and the molecular mechanism of promoting metabolism have not been fully explored.

Herein, we established an HFD-induced obesity model for C57BL/6J mice designed to evaluate the ameliorating potential of *Bifidobacterium longum* subsp. *infantis* FB3-14 (FB3-14), a novel probiotic strain isolated from breastfed infant feces. Several aspects, including weight comparison, histopathology, serum lipid level, hepatic function, and lipid-metabolism-related gene expression profiling, were investigated to assess the protective effects. Moreover, 16S ribosomal RNA (rRNA) sequencing was used to explore the effect of FB3-14 on the gut microbial population, thus identifying the role of intestinal microecology against obesity mediating FB3-14. Our study aimed to provide an alternative nutrition strategy for prophylaxis and treating obesity and related metabolic diseases.

## 2. Materials and Methods

### 2.1. Probiotic Strain

The probiotic strain *Bifidobacterium longum* subsp. *infantis* FB3-14 (FB3-14) used in this study was screened from the feces of breastfed healthy infants. The probiotic properties and safety of this strain were evaluated, as shown in Figure S1, and it is recognized as a safe and edible strain. FB3-14 was stored in the General Microbiology Center of China Microbiological Culture Preservation Management Committee (CGMCC) with preservation number No. 25762. The strain was strictly anaerobically cultured at 37 °C in an anaerobic workstation using modified MRS broth/agar medium (de Man, Rogosa and Sharpe) supplemented with 0.05% (*w*/*v*) l-cysteine hydrochloride.

### 2.2. Animal Experiments

The specific-pathogen-free C57BL/6J mice (4–6 weeks, male) were purchased from Beijing Vital River Laboratory Animal Technology Co., Ltd. (Beijing, China) and housed in the Nankai University Laboratory Animal Center under controlled conditions (temperature, 20–25 °C; humidity, 50–60%; 12/12 h light/dark cycle). After acclimatization for one week, all mice were randomly divided into 3 groups (*n* = 8), as follows: the normal diet control (CON) group (3.44 kcal/g, 10% energy from fat), the high-fat-diet model (HFD) group (4.23 kcal/g, 60% energy from fat), and the HFD + FB3-14 treatment (FB3-14) group (4.23 kcal/g, 60% energy from fat, supplemented with *Bifidobacterium longum* subsp. *infantis* FB3-14 1 × 10^9^ CFU/mouse/day). The composition and energy-supplying ratio of each diet are shown in Tables S1 and S2. The mice were allowed access to food and water ad libitum throughout the study. Body weights of mice were measured weekly, and food intake was also monitored. After 8-week treatment, three groups of mice were anesthetized and sacrificed to collect serum, liver, epididymal fat, cecal contents, and colon.

### 2.3. Serum Biochemical Analysis

For the determination of hyperlipidemic markers in the blood, the levels of total cholesterol (TC), triglyceride (TG), low-density lipoprotein cholesterol (LDL-C), and high-density lipoprotein cholesterol (HDL-C) were determined using Nanjing Jiancheng Institute of Bioengineering ELISA kits (Nanjing, China). Liver function indicator (serum aspartate transaminase (AST) and alanine transaminase (ALT)) and gut-secreted hormone (glucagon-like peptide-1 (GLP-1) and peptide YY (PYY)) levels were determined using Nanjing Jiancheng Institute of Bioengineering ELISA kits (Nanjing, China). On the last day of the 7th week, fasting blood glucose (FBG) levels of different groups of mice were measured after 16 h of fasting.

### 2.4. Histological Examination

The tissues were fixed in 10% neutral-buffered formalin, followed by paraffining, embedding and sectioning, hematoxylin–eosin (H&E) staining, and microscopic examination to complete the pathological evaluation. The adipocyte area was quantified using ImageJ 1.54 software. The histological scoring criteria for the liver tissue are shown in Table S3.

### 2.5. Quantification of Gene Expression

Previous research methods were used [16]. Briefly, the RNA was extracted using TriQuick reagent and reverse-transcribed into cDNA. The mRNA-expression-level-related genes were examined using RT-qPCR technique. The specific primer sequences applied are shown in Table S4. Relative quantification was normalized using *β-actin* control and calculated in accordance with the comparative 2^−∆∆Ct^ method.

### 2.6. Short-Chain Fatty Acid (SCFAs) Determination

SCFAs in cecal content were determined as previous instructions [17]. In brief, about 0.2 g of fresh fecal samples was homogenized with 1 mL ultra-pure water and two steel balls, followed by centrifugation (12,000× *g*, 5 min) to collect the supernatant. Then, 100 μL 10% sulfuric acid was added to acidize, and 0.5 mL ethyl ether was added to extract SCFAs. The upper layer of organic phase after static extraction was filtered using 0.22 μm nylon membranes and collected in 2 mL brown autosampler vials for detection. DB-FFAP capillary column (30 m × 0.25 mm i.d., 0.25 μm, Agilent, Santa Clara, CA, USA) was performed with gas chromatography to quantify SCFAs in our study. The single standard solution was injected first, with the relative peak time recorded. Then, the standard mixtures of different concentrations were injected, and the relative peak area was recorded for the construction of standard curves. Different groups of samples were injected finally, and the concentration of each sample was calculated.

### 2.7. Gut Microbiota Profiling

Total cecal content DNA was extracted and qualified. The V3-V4 regions of the bacterial 16S rDNA genes were amplified by PCR using the primers 338F: 5′-ACTCCTACGGGAGGCAGCA-3′ and 806R: 5′-GGACTACHVGGGTWTCTAAT-3′. Qiime (version 1.9.1) was used to estimate alpha diversity within samples and beta diversity between samples. A principal coordinate analysis (PCoA) based on the bray_curtis distance algorithm of the OTUs was performed to investigate the β diversity. Differentially abundant taxa were determined using linear discriminant analysis effect size (LEfSe). Spearman correlation analysis was conducted to explore the correlation between intestinal microbiota and metabolic parameters.

### 2.8. Statistical Analysis

Prism 8.0.1 (GraphPad, La Jolla, CA, USA) was used for experimental data analysis, and all the results were presented as the mean ± SD. Statistical analysis of different groups was performed using one-way analysis of variance (ANOVA), Dunnett’s multiple comparison tests, and Kruskal–Wallis test for liver histological score. Statistical significance was set at * *p* < 0.05, ** *p* < 0.01, or *** *p* < 0.001.

## 3. Results

### 3.1. FB3-14 Inhibited Overweight and Dyslipidemia

**Figure 1:**
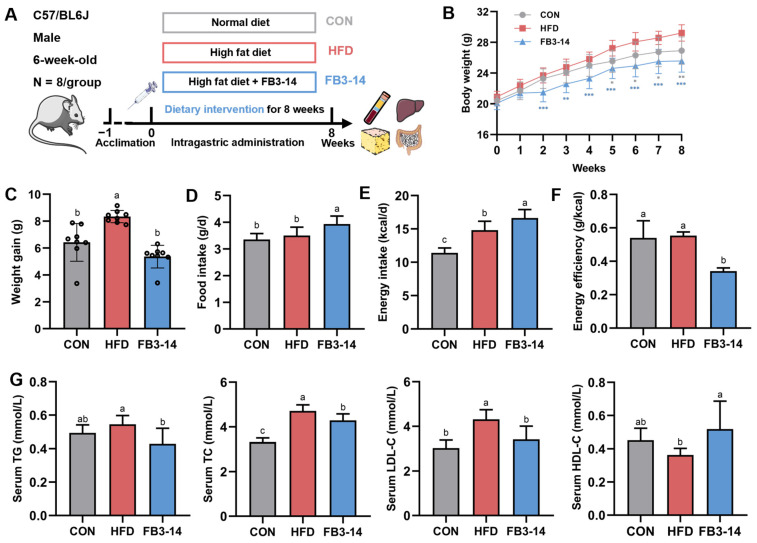
FB3-14 inhibited overweight and dyslipidemia in high-fat-diet-fed mice. (**A**) Schematic diagram of C57/BL6J mice high-fat diet induction and FB3-14 intervention model. (**B**) Body weight during the entire experimental period. (**C**) Weight gain after 8-week intervention. (**D**) Average daily food intake. (**E**) Average daily energy intake. (**F**) Energy efficiency. (**G**) Serum TG, TC, LDL-C, and HDL-C levels. Data are shown as the mean ± SD (*n* = 8). (**B**) * *p* < 0.05, ** *p* < 0.01, and *** *p* < 0.001 versus the HFD group. (**C**–**G**) The mean value with different letters indicates significant differences (*p* < 0.05). CON: control; HFD: high-fat-diet-fed mice; FB3-14: high-fat-diet-fed mice treated with *Bifidobacterium longum* subsp. *infantis* FB3-14.

After one week of acclimatization, obesity was successfully induced by continuous HFD feeding of C57/BL6J mice for the next 8 weeks (Figure 1A). The body weight of mice in the HFD group was significantly distinguished from that of CON group mice starting in the 5th week, while FB3-14 treatment suppressed the dramatic rise (Figure 1B). At the end of the treatment, the FB3-14 group mice showed prominently lower weight gain (Figure 1C), even though the mice in this group consumed more food and energy per day (Figure 1D,E). The energy efficiency comparison suggested that *Bifidobacterium longum* subsp. *infantis* FB3-14 alleviated HFD-induced overweight, mainly by decreasing energy efficiency (Figure 1F).

Additionally, serum lipid indicators proved that HFD caused dyslipidemia in obese mice (Figure 1G). Conversely, FB3-14 significantly inhibited the obvious increase in serum TC and LDL-C levels and reversed the abnormal changes in serum TG and HDL-C in HDF-fed mice to a certain extent. To summarize, these results preliminarily demonstrate that FB3-14 effectively reduced HFD-induced overweight and dyslipidemia in mice.

### 3.2. FB3-14 Alleviated Lipid Accumulation and Lipid Metabolism Disorders

**Figure 2:**
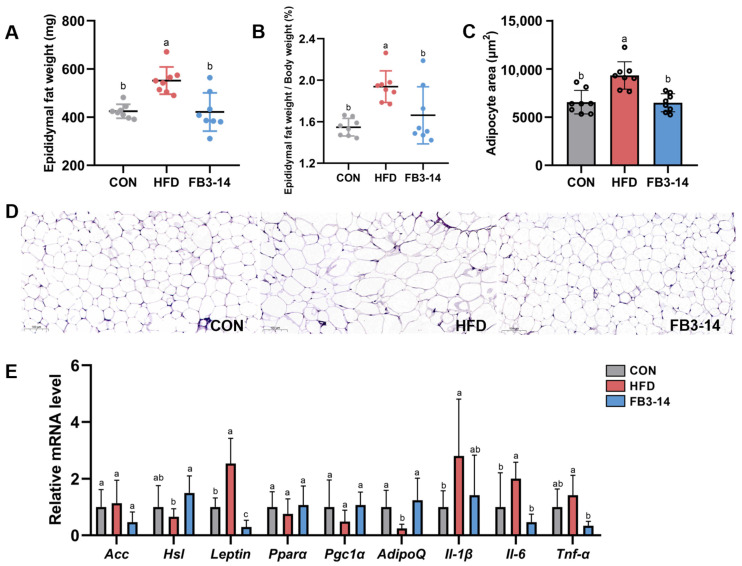
FB3-14 alleviated lipid accumulation and lipid metabolism disorders in high-fat-diet-fed mice. (**A**) Epididymal fat weight. (**B**) Epididymal fat/body weight ratio. (**C**) Adipocyte area. (**D**) Representative histologic images of hematoxylin and eosin (H&E) staining of epididymis adipose sections. (**E**) Relative mRNA expression of *Acc*, *Hsl*, *Leptin*, *Pparα*, *Pgc1α*, *AdipoQ*, *Il-1β*, *Il-6*, and *Tnf-α* in the epididymal adipose tissue. Data are shown as the mean ± SD (*n* = 8). The mean value with different letters indicates significant differences (*p* < 0.05). CON: control; HFD: high-fat-diet-fed mice; FB3-14: high-fat-diet-fed mice treated with *Bifidobacterium longum* subsp. *infantis* FB3-14.

HFD-fed mice had remarkably higher epididymal fat weight than the other two groups (Figure 2A). The epididymal fat weight/body weight ratio was calculated to evaluate the degree of obesity in mice. As shown in Figure 2B, the proportion of epididymal fat weight to body weight of mice in the HFD group was prominently increased, while it was significantly decreased after the intervention of FB3-14. Histopathological observation of epididymal fat visually revealed the excessive adipocyte expansion caused by HFD and the significant inhibitory effect of FB3-14 intervention on this pathological lesion (Figure 2C,D). Further, to explore the roles of FB3-14 on lipid metabolism in HFD-induced obese mice, mRNA expression levels of relevant indicators were measured in the experiment. Two genes, *Leptin* and *AdipoQ* in the epididymal fat of HFD group mice, were shown to vary significantly after 8-week treatment, and this effect was prevented by FB3-14 (Figure 2E). Moreover, HFD also caused a significant upregulation of inflammation-related mRNA expression in epididymal fat, but the gene expression of *Il-6* and *Tnf-α* was effectively decreased due to the effects of FB3-14 (Figure 2E).

Taken together, these results suggest that the FB3-14 intervention remarkably inhibited the lipid accumulation and aberrant expression of obesity-related genes in HFD-fed mice.

### 3.3. FB3-14 Ameliorated Impaired Liver Function and Systemic Low-Grade Inflammation

**Figure 3:**
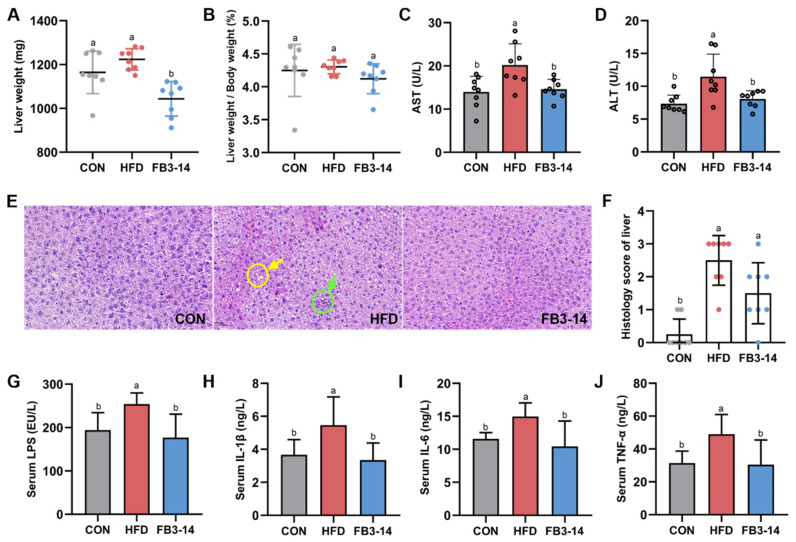
FB3-14 ameliorated impaired liver function and systemic low-grade inflammation in high-fat-diet-fed mice. (**A**) Liver weight. (**B**) Liver/body weight ratio. (**C**) Serum AST levels. (**D**) Serum ALT levels. (**E**) Representative histologic images of hematoxylin and eosin (H&E) staining of liver sections. (**F**) Histology score of liver. (**G**) Serum LPS levels. (**H**) Serum IL-1 levels. (**I**) Serum IL-6 levels. (**J**) Serum TNF-α levels. Data are shown as the mean ± SD (*n* = 8). The mean value with different letters indicates significant differences (*p* < 0.05). CON: control; HFD: high-fat-diet-fed mice; FB3-14: high-fat-diet-fed mice treated with *Bifidobacterium longum* subsp. *infantis* FB3-14.

Mice in the FB3-14 group had lower liver weight, while HFD and FB3-14 treatments had no significant effect on the liver weight/body weight ratio in mice generally (Figure 3A,B). Serum ALT and AST are important indicators of liver function [18]. FB3-14 intervention significantly inhibited the abnormal elevation of ALT and AST levels in obese mice (Figure 3C,D). The histological section of liver tissue in Figure 3E,F shows the hepatocyte morphology and scores of liver tissue in different groups of mice. Steatosis, vacuoles formed after the dissolution of lipid droplets (yellow arrow), accompanied by a small quantity of inflammatory infiltration (green arrow), was observed in hepatocytes of the HFD group mice compared to the CON group. FB3-14 intervention ameliorated hepatocyte swelling and cytoplasmic boundary indistinction caused by HFD in obese mice. Furthermore, LPS has been identified as a triggering factor for inflammatory cytokines, which lead to metabolic diseases such as obesity [19]. HFD significantly increased serum LPS levels and pro-inflammatory factors, including IL-6, IL-1β, and TNF-α, in obese mice, while these markers were significantly decreased in the serum of the FB3-14 treatment mice (Figure 3G–J).

Taken together, these results suggest that FB3-14 intervention alleviated hepatic steatosis and liver function impairment in HFD-fed mice, as well as suppressing the development of HFD-driven systemic low-grade inflammation.

### 3.4. Effects of FB3-14 on Intestinal Flora Structure Disturbance

**Figure 4:**
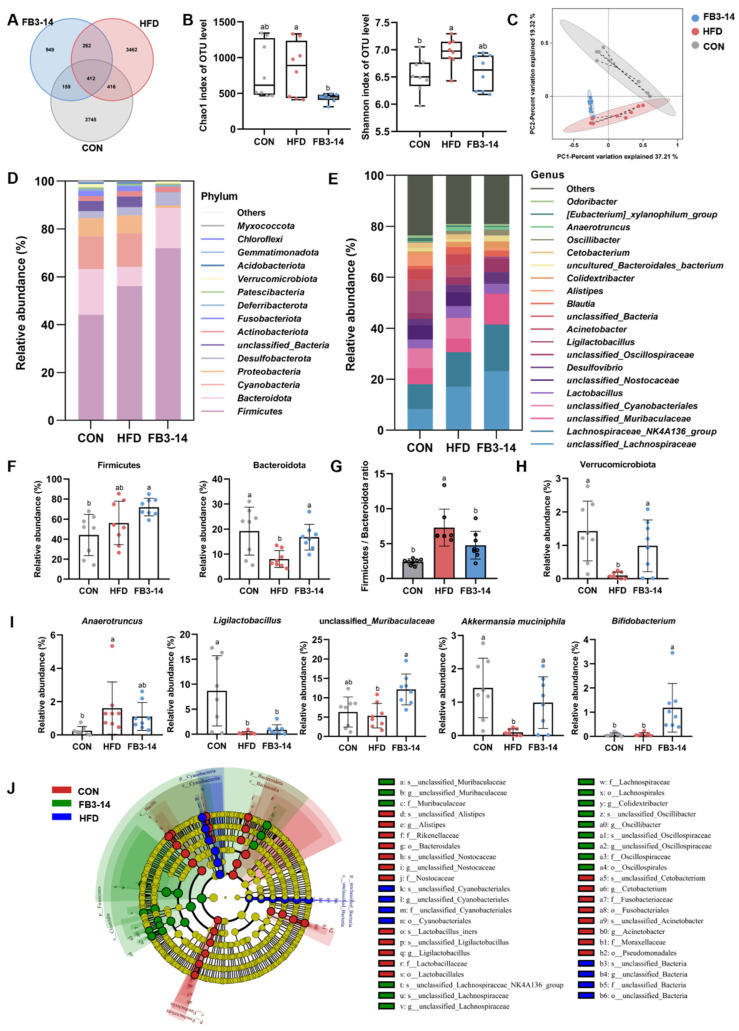
Effects of FB3-14 on intestinal flora structure disturbance in high-fat-diet-fed mice. (**A**) Venn diagrams of groups. (**B**) Chao1 index and Shannon index. (**C**) PCoA. (**D**) Bacterial community structure at the phylum level. (**E**) Bacterial community structure at the genus level. (**F**) Relative abundance of Firmicutes and Bacteroidota. (**G**) Firmicutes/Bacteroidetes ratio. (**H**) Relative abundance of Verrucomicrobiota. (**I**) Relative abundance of the significantly differential flora at the genus level. (**J**) Branch diagram depicting the output of the LEfSe analysis. Data are shown as the mean ± SD (*n* = 8). The mean value with different letters indicates significant differences (*p* < 0.05). CON: control; HFD: high-fat-diet-fed mice; FB3-14: high-fat-diet-fed mice treated with *Bifidobacterium longum* subsp. *infantis* FB3-14.

The Venn diagram showed the composition of the intestinal microbiota in response to different dietary interventions. The CON, HFD, and FB3-14 groups resulted in 3745, 3462, and 949 specific operational taxonomic units (OTUs), respectively, and shared 412 overlapping OTUs for clustering all high-quality sequences with 97% identity (Figure 4A). The Chao1 index and Shannon index were used to evaluate the alpha diversity of intestinal microbes in different groups of mice. Figure 4B showed that HFD caused a significant increase in the Shannon index, the value of which decreased after FB3-14 intervention. After eight weeks of treatment, the Chao1 index of the FB3-14 group was significantly lower than the HFD group. In addition, beta diversity was assessed by PCoA, which showed clear clustering at the OTU level among the healthy mice, obese mice, and FB3-14-treated mice. The separation of the three groups demonstrated the shifts in microbial structure caused by HFD and implicated the dramatic impact of FB3-14 on the changes in microbial composition (Figure 4C).

To further explore the preventive effects and potential mechanisms of FB3-14 on HFD-induced obesity, we present a histogram of the relative richness histogram of the top 10/15 annotated species at the phylum/genus level in Figure 4D,E, respectively. At the phylum level, Firmicutes and Bacteroides were the dominant phyla in all three groups, accounting for 44.11% and 19.21% of the microbial profiles in the CON group, but significantly changed to 56.13% and 8.05% after HFD treatment (Figure 4F). Strikingly, FB3-14 effectively reversed and normalized the Firmicutes/Bacteroides ratio imbalance caused by HFD (Figure 4G). Moreover, FB3-14 restored the markedly reduced abundance of Verrucomicrobiota in HFD mice (Figure 4H). Furthermore, the gut microbiota at the genus level were different in each group. HFD caused significant variation in *Anaerotruncus*, *Ligilactobacillus*, and *Akkermansia muciniphila* abundance, while FB3-14 intervention prominently enhanced the abundance of *unclassified_Muribaculaceae*, *Akkermansia muciniphila*, and *Bifidobacterium* compared to the HFD group (Figure 4I). LEfSe analysis was used to taxonomize specific phylogenetic types significantly related to the intervention and analyze intestinal microorganism biomarkers in the three groups. As illustrated in Figure 4J, the bacteria from the genera of *unclassified_Cyanobacteriales* and *unclassified_Bacteria* were enriched in the HFD group, and higher levels of *unclassified_Muribaculaceae*, *Lachnospiraceae_NK4A136_group*, and *unclassified_Oscillospiraceae* were observed in the FB3-14 group.

In summary, these results suggest that FB3-14 modified metabolic disorders in HFD-fed mice by reprogramming the gut microecology.

### 3.5. FB3-14 Restored Lipid Metabolism by Upregulating Butyric Aid Content

**Figure 5:**
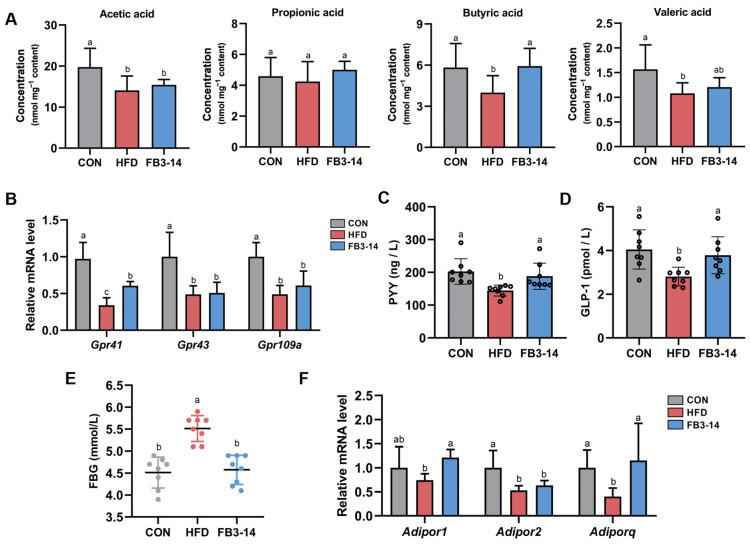
FB3-14 restored lipid metabolism in high-fat-diet-fed mice by upregulating butyric acid content. (**A**) Concentration of acetic acid, propionic acid, butyric acid, and valeric acid in mice cecal contents. (**B**) Relative mRNA expression of *Gpr41*, *Gpr43*, and *Gpr109a*. (**C**) Serum PYY levels. (**D**) Serum GLP-1 levels. (**E**) Fasting blood glucose (FBG) concentration. (**F**) Relative mRNA expression of *Adipor1*, *Adipor2*, and *Adiporq*. Data are shown as the mean ± SD (*n* = 8). The mean value with different letters indicates significant differences (*p* < 0.05). CON: control; HFD: high-fat-diet-fed mice; FB3-14: high-fat-diet-fed mice treated with *Bifidobacterium longum* subsp. *infantis* FB3-14.

As one of the metabolites of gut microbes, SCFAs play an indispensable role in maintaining gut and metabolic health [20]. HFD resulted in an obvious decrease in acetic acid, butyric acid, and valeric acid in the cecum contents of mice, but FB3-14 intervention significantly upregulated their butyric acid levels (Figure 5A). As expected, compared with the CON group, the mRNA relative expression levels of SCFA receptors GPR41, GPR43, and GPR109A in the HFD group were significantly reduced, while FB3-14 intervention mitigated the damage to GPR41 (Figure 5B). Since butyric acid increases the expression of PYY and GLP-1 by GPR41 and GPR43 in the colon [21], we measured the serum levels of PYY and GLP-1. The results showed that PYY and GLP-1 levels in the FB3-14 group were noteworthily higher than those in the HFD group (Figure 5C,D). Meanwhile, FB3-14 intervention also prominently suppressed fasting blood glucose concentrations in HFD mice (Figure 5E). Further, the relative mRNA expression levels of *Adiporq* and *Adipor2* in HFD-fed mice were found to be significantly decreased, while FB3-14 effectively upregulated the relative expression level of *Adipor1* and restored the expression of *Adiporq* (Figure 5F).

Taken together, these results suggest that FB3-14 intervention increased GPR41 levels associated with higher levels of butyric acid content, increased the PYY and GLP-1 levels, and upregulated the mRNA relative expression of *Adipor1* and *Adiporq*, thereby alleviating HFD-induced obesity.

### 3.6. Correlation Analysis between Intestinal Microbiota and Metabolic Parameters

**Figure 6:**
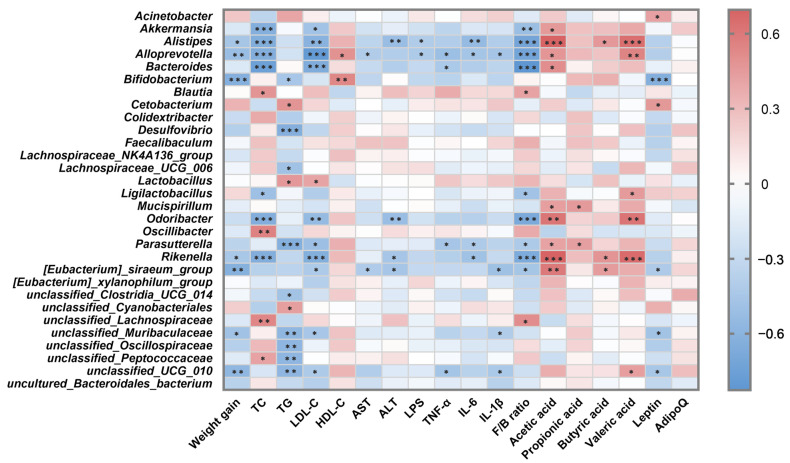
Correlation analysis between intestinal microbiota and metabolic parameters. * *p* < 0.05, ** *p* < 0.01, *** *p* < 0.001.

To identify specific microbial strains that may mediate FB3-14 in alleviating obesity, Spearman’s correlation analysis was used to evaluate the correlation between gut microbes and obesity-related indicators, as shown in the heatmap (Figure 6). The 30 most abundant OTUs were selected for analysis, of which 25 OTUs were relevant to at least one obesity metabolism index. *Bifidobacterim*, *unclassified_Muribaculaceae*, and *unclassified_UCG_010* were negatively associated with body weight gain and levels of inflammatory factors TNF-α and IL-1β. Moreover, *Akkermansia*, *Alistipes*, and *Rikenella* were significantly negatively correlated with serum TC and LDL-C levels. These results suggest that these intestinal bacteria negatively related to obesity phenotypes might be the valuable bacterial genera conducive to FB3-14’s anti-obesity effects.

## 4. Discussion

With the progress and development of society, plenty of precipitating factors such as diets, sedentary lifestyle, mental stress, and genetic inheritance may induce lipid metabolism disorders, thereby causing obesity and other related metabolic diseases, including type 2 diabetes, CVD, and NAFLD [22]. The gut microbiome and its metabolites have been shown to affect energy homeostasis and have emerged as key regulators of host metabolism [23]. Therefore, increasing studies have emphasized suppressing abnormal lipid accumulation by targeting gut microbiota, thus attenuating obesity symptoms. As a functional food, probiotics represented by *Lactobacillus* and *Bifidobacterium* can exert health benefits by rapidly and reproducibly altering the gut microbiota [24]. Until now, probiotics have been considered novel and effective anti-obesity treatments [25,26], while the studies on mechanisms of *Bifidobacterium longum* subsp. *infantis* alleviating obesity is still limited.

In our study, an HFD-induced obese model was established to evaluate the potential of *Bifidobacterium longum* subsp. *infantis* FB3-14 in normalizing lipid glucose metabolism disorders and preventing obesity. First, the lower weight growth rate and adipose/body weight ratio of FB3-14 group mice intuitively confirmed the anti-obesity ability of FB3-14. Meanwhile, FB3-14 supplementation significantly improved diet-induced metabolic syndrome lipid profile markers and fasting blood glucose. These results were in line with other previous animal experiments aiming at probiotics, including *Lactobacillus kefiri* DH5, *Lactobacillus paracasei*, *Bifidobacterium animalis* 01, and *Bifidobacterium* sp. MKK4 showed consistent results in inhibiting lipid metabolism disorders by reducing serum TC, TG, and LDL-C and raising HDL-C levels in mice/rats [27,28,29,30].

Adipose tissue and the liver are essential organs for lipid metabolism as the main sites for the release of enzymes and adipokines concerned with the processes of adipogenesis, lipogenesis, and lipolysis [16]. FB3-14 intervention was found to effectively alleviate HFD-induced histological damage, including the abnormal expansion of epididymal adipocytes as well as steatosis and low-grade inflammation in hepatocytes, while the underlying molecular mechanisms were worthy of further investigation. Fat tissue secretes many adipokines and constitutes an extremely active endocrine organ [31]. These adipokines include hormones (such as leptin and adiponectin), cytokines, growth factors, and vasoactive factors, which not only affect adipobiology and function but are also released into the blood to regulate numerous biological processes [32]. Notably, our results showed that FB3-14 prominently upregulated the relative expression level of the gene *Hsl*, an enzyme associated with lipolysis. Lipids are stored primarily in the form of triacylglycerol (TAG) in mature white fat cells; hormone-sensitive lipase (HSL) is involved in the hydrolysis process of hydrolyze diglycerol (DAG), although it also has TAG hydrolyzing activity [33]. Previous studies also found that probiotics, *Lactobacillus sakei* and *Lactobacillus plantarum HAC01*, improved adipose tissue dysfunction by upregulating lipolytic-enzyme-related genes or downregulating genes encoding lipogenic enzymes [34,35]. FB3-14 also prominently inhibited the abnormal expression of leptin and adiponectin genes induced by HFD. Leptin signaling regulates the sympathetic structure of adipose tissue via a top-down neural pathway and is crucial for energy homeostasis [36]. Leptin is secreted by adipose tissue, and its content in serum is positively related to the size of adipose tissue [37], which was also observed in our study. Low levels of adiponectin signaling lead to severe insulin resistance [38], while FB3-14 intervention enhanced the expression of the *AdipoQ* gene in fat tissue significantly. Moreover, impaired liver function homeostasis and low-grade systemic inflammation are closely associated with the development of obesity and other metabolic diseases. One study showed that locally produced inflammatory mediators, especially tumor necrosis factor (TNF), regulate basal lipolysis through specific signal transduction pathways [39]. FB3-14 intervention relieved hepatocyte damage and systemic low-grade inflammation in obese mice by reducing AST, ALT, and pro-inflammatory factor levels in serum and adipose tissue.

The findings of Fredrik Backhed et al. suggested that the gut microbiome influenced energy harvest from the diet and energy storage in the host [3]. Primarily, numerous studies, including our experiment, found that long-term HFD intervention induction increased the Firmicutes/Bacteroidetes (F/B ratio) in obese individuals [40]. However, the FB3-14 treatment significantly reduced the F/B ratio in HFD-fed mice. Shanthi G Parkar et al. also found that polyphenols stimulated the proliferation of *Bifidobacterium* spp., decreased the F/B ratio, and facilitated SCFA production, demonstrating an association between bifidobacteria and anti-obesity effects [41]. Next, by comparing the intestinal microbiota structure of mice in different intervention groups, at the genus level, the abundance of *Anaerotruncus*, *Ligilactobacillus*, *Akkermansia muciniphila*, *Rikenella*, *unclassified_Muribaculaceae*, *Lachnospiraceae_NK4A136_group*, and *Bifidobacterim* showed significant difference in this study. As in previous studies, we also found that the abundance of the opportunistic pathogen *Anaerotruncus* was amplified in HDF-fed mice [42]. Importantly, FB3-14 intervention remarkably reversed the reduction in *Akkermansia muciniphila* and *unclassified_Muribaculaceae.* Studies have shown that the relative abundance of probiotic *Akkermansia muciniphila* was low in obese and diabetic mice, indicating that *Akkermansia muciniphila* was negatively correlated to the pathogenesis of obesity [43]. Moreover, *Muribaculaceae* was also shown to suppress obesity by participating in mitochondrial energy metabolism and inflammatory processes [44].

On the other hand, in part, HFD induces obesity by affecting the levels of calculating microbial metabolites, which can contribute to alterations in satiety, energy metabolism, lipid accumulation, and systemic inflammation [45]. In our experiments, FB3-14 treatment was found to significantly upregulate butyric acid content in HFD-fed mice, accompanied by the rising level of GPR41. Butyrate, which is produced by some species from *Ruminococcaceae*, *Lachnospiraceae*, *Erysipelotrichaceae*, and *Clostridiaceae*, was reported to increase the expression of PYY and GLP-1 in the colon by activating endogenous GPR41 and 43 and improve glucose homeostasis [21,46]. PYY and GLP-1, the gastrointestinal hormones synthesized and secreted by enteroendocrine L cells in the distal intestine, are implicated in appetite, gut motility, and insulin regulation [47]. Additionally, after binding to GPR41/43, SCFAs were found to play an anti-inflammatory role by inhibiting the activation of NF-κB in the host immune cells [45,48]. The remission of inflammation was also found in our study by observing the decreasing levels of proinflammatory factors in the serum and livers of mice. Overall, FB3-14 treatment might mitigate obesity and inflammation by mediating GPR41 to upregulate the serum PYY and GLP-1 levels by increasing the butyric acid content.

In our study, FB3-14 was observed to relieve the HFD-induced adverse influences on lipid metabolism and intestinal microbiota. Our results might provide a theoretical basis for the preventive potentials of *Bifidobacterium longum* subsp. *infantis* FB3-14 on obesity and related metabolic diseases. Nevertheless, there is still a need for long-term clinical verifications to confirm its efficacy in distinguished dietary backgrounds.

## 5. Conclusions

In our study, the positive anti-obesity effects of the strain *Bifidobacterium longum* subsp. *infantis* FB3-14 ameliorated abnormal weight gain, dyslipidemia, lipid accumulation, liver function impairment, low-grade systemic inflammation, and differential expression of genes related to lipid metabolism in HFD-fed mice. Moreover, FB3-14 treatment also reprogrammed the intestinal microbial composition; promoted the abundance of *Akkermansia muciniphila*, *unclassified_Muribaculaceae*, *Lachnospiraceae_NK4A136_group*, and *Bifidobacterim*, which were inversely associated with obesity-related parameters; and might enhance the secretion of PYY and GLP-1 by upregulating the butyric acid level mediating GPR41. Our findings provide evidence that the novel strain *Bifidobacterium longum* subsp. *infantis* FB3-14 can inhibit HFD-induced overweight and relieve gut microbiome dysregulation and may also offer a new nutritional strategy for alleviating obesity and related metabolic diseases.

## Data Availability

Not applicable.

## Supplementary Materials

The following supporting information can be downloaded at https://www.mdpi.com/article/10.3390/nu15194104/s1, Figure S1: Safety evaluations of *Bifidobacterium longum subsp. infantis* FB3-14. (A) Hemolysis Assays. (B) Determination of nitrate reductase activity. (C) Determination of amino acid decarboxylase activity. FB3-14: *Bifidobacterium longum subsp. infantis* FB3-14. Table S1: Primer sequences for qPCR. Table S2: Mass Ratio and Energy Supply Ratio. Table S3: Histological Score of Liver Tissue. Table S4: Specific Primer Sequences for RT-qPCR.
