# Supplementary material for: Ameliorating Effects of Bifidobacterium longum subsp. infantis FB3-14 against High-Fat-Diet-Induced Obesity and Gut Microbiota Disorder
Source: Nutrients. 2023 Sep 22;15(19):4104. doi: 10.3390/nu15194104 (PMC10574542; doi:10.3390/nu15194104)
Supplement: Supplementary file 1 [file nutrients-15-04104-s001.zip › nutrients-2610339-supplementary.pdf]

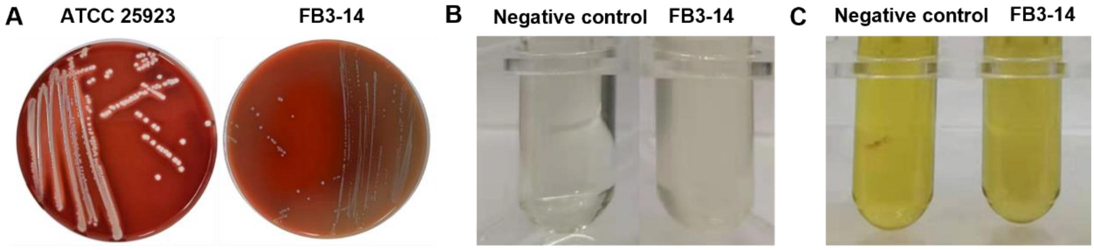

Figure S1. Safety evaluations of *Bifidobacterium longum subsp. infantis* FB3-14. (**A**) Hemolysis Assays. (**B**) Determination of nitrate reductase activity. (**C**) Determination of amino acid decarboxylase activity. FB3-14: *Bifidobacterium longum subsp. infantis* FB3-14.

Table S1. Diets Formula.

Compositions	Normal Diet	High Fat Diet (HFD)
Casein	800 kcal/kg	800 kcal/kg
L-Cystine	12 kcal/kg	12 kcal/kg
Corn Starch	2024.8 kcal/kg	0
Maltodextrin	500 kcal/kg	500 kcal/kg
Sucrose	275 kcal/kg	275 kcal/kg
Soybean Oil	225 kcal/kg	225 kcal/kg
Lard	180 kcal/kg	2205 kcal/kg
Vitamin Mix	40 kcal/kg	40 kcal/kg

Table S2. Mass Ratio and Energy Supply Ratio.

Energy-Supplying Substance	Normal Diet			High Fat Diet (HFD)		
	Mass Ratio, %	Kcal/kg	Energy Supply Ratio, %	Mass Ratio, %	Kcal/kg	Energy Supply Ratio, %
Protein	18.8	752	20.54	23.25	930	18.14
Fat	5.2	468	12.79	34.55	3110	60.65
Carbohydrate	61.0	2440	66.67	27.20	1088	21.22
Total energy		3660	100		5128	100

Table S3. Histological Score of Liver Tissue.

Scores	Histological Findings
0	No fibrosis
1	Fibrous portal expansion
2	Periportal fibrosis and Occasional septa
3	Many septa and Architectural distortion
4	Probable/Definite cirrhosis

Table S4. Specific Primer Sequences for RT-qPCR.

Genes	Forward Primer (5′→3′)	Reverse Primer (5′→3′)
β-actin	GGCTGTATTCCCCTCCATCG	CCAGTTGGTAACAATGCCATGT
Acc	GGCAGCAGTTACACCACATAC	TCATTACCTCAATCTCAGCATAGC
Hsl	GCTAGCCAGGCTCATCTCCT	GTTCTTGAGGTAGGGCTCGT
Leptin	CCTGTGGCTTTGGTCCTATCTG	AGGCAAGCTGGTGAGGATCTG
Pparα	TGCAGCCTCAGCCAAGTTGAA	TCCCGAACTTGACCAGCCA
Pgc1α	AGCCGTGACCACTGACAACGAG	GCTGCATGGTTCTGAGTGCTAAG
Adiporq	AGGCCGTTCTCTTCACCTAC	TACACCTGGAGCCAGACTTG
Adipor1	CTATCGCTGAGGGCTTTGTC	CAGGAATCCGAGCAGCATAC
Adipor2	ACGATGTGCTACCGGATTGG	AAACAGGCCCGAAAGGAAGG
Il-1β	AAGGGCTGTTCCAAACCTTTGAC	TGCCTGAAGCTTTGTTGATGTGC
Il-6	TAGTCCTTCCTACCCCAATTTCC	TTGGTCCTTAGCCACTCCTTC
Tnf-α	AATGGCCTCCCTCTCATCAG	CCACTTGGTGGTTTGCTACG
Gpr-41	CAAGTTCCAAGCCGACTTTC	TGGATGGCTCTTCTCCATTC
Gpr-43	GAGGCTGTGGTGTTCAGTTC	GCATAGAGGAGGCAGGATTG
Gpr-109a	GGCACGATGCTATGTTCCTC	GATGGCCCTCTTGATCTTGG

Acc, acetyl-CoA carboxylase; *Hsl*, hormone-sensitive lipase; *Pparα* peroxisome proliferator activated receptor alpha; *Pgc1α*, peroxisome proliferator-activated receptor c coactivator 1*α*; *Adiporq*, adiponectin; *Adipor1*, adiponectin receptor 1; *Adipor2*, adiponectin receptor 2; *Il-1β*, interleukin-1β; *Il-6*, interleukin-6; *Tnf-α*, tumor necrosis factor *α*; *Gpr-41*, G protein coupling receptors 41.
